# Ultrasound-guided peripheral vascular catheterization in pediatric patients: a narrative review

**DOI:** 10.1186/s13054-020-03305-7

**Published:** 2020-09-30

**Authors:** Yoshinobu Nakayama, Jun Takeshita, Yasufumi Nakajima, Nobuaki Shime

**Affiliations:** 1 Department of Anesthesiology and Critical Care, Kyoto Prefectural University of Medicine, Kyoto, 602-8566 Japan; 2 Department of Molecular, Cellular and Biomedical Sciences, CUNY School of Medicine, City College of New York, New York, USA; 3 Department of Anesthesiology, Osaka Women’s and Children’s Hospital, Osaka, Japan; 4 Department of Anesthesiology and Critical Care, Kansai Medical University, Osaka, Japan; 5 Outcomes Research Consortium, Cleveland, OH USA; 6 Department of Emergency and Critical Care Medicine, Graduate School of Biomedical & Health Sciences, Hiroshima University, Hiroshima, Japan

**Keywords:** Ultrasound, Pediatrics, Peripheral catheterization, Blood vessels

## Abstract

Peripheral vascular catheterization (PVC) in pediatric patients is technically challenging. Ultrasound guidance has gained the most interest in perioperative and intensive care fields because it visualizes the exact location of small target vessels and is less invasive than other techniques. There have been a growing number of studies related to ultrasound guidance for PVC with or without difficult access in pediatric patients, and most findings have demonstrated its superiority to other techniques. There are various ultrasound guidance approaches, and a comprehensive understanding of the basics, operator experience, and selection of appropriate techniques is required for the successful utilization of this technique. This narrative review summarizes the literature regarding ultrasound-guided PVC principles, approaches, and pitfalls to improve its clinical performance in pediatric settings.

## Background

Peripheral vascular catheterization (PVC) is an essential skill for perioperative, intensive, and emergency healthcare providers. In small children, operators often misjudge the exact location of vessels due to the presence of thick subcutaneous tissue and smaller vessel diameters, which in turn makes catheterization challenging. Currently, ultrasound guidance is an approach majorly used in such difficult cases.

Numerous studies have demonstrated the utility of an ultrasound-guided approach in PVC in pediatric patients. Systematic reviews and meta-analyses have reported that ultrasound guidance improves pediatric PVC in terms of success rate, procedure time, number of attempts, and number of complications [1–4]. Regarding infants and small children, ultrasound guidance increased the first-attempt rate, up to a relative risk of 2.2 in radial arterial catheterization, than the other techniques [3]. In neonates, it also improved first-attempt success rate, up to a relative risk of ~ 4.0 [5]. Thus, the utility of ultrasound guidance is considered higher in smaller children.

However, ultrasound-guided PVC in smaller children remains challenging for less-experienced operators. The first-attempt success rate of ultrasound-guided radial arterial catheterization in pediatric patients was significantly increased than the palpation technique when performed by operators who were familiar with ultrasound guidance [3]; therefore, well-defined training based on a sufficient understanding of ultrasound guidance in pediatric settings has been recommended [3, 6].

This narrative review summarizes the accumulated knowledge and experiences to aid and improve ultrasound-guided PVC in pediatric patients. Notably, we proposed seven basic questions that arise while performing the procedure (Table 1). We then intensively searched bibliographic databases and summarized the results to answer these questions.

**Table 1:** Seven clinical questions regarding ultrasound-guided peripheral vascular catheterization in pediatric patients

Clinical questions	
1. Where is the possible access site?	
2. How do we check the vascular condition?	
3. How do we obtain the optimal position for the procedure?	
4. How do we determine the optimal catheter size?	
5. What are the major approaches available for ultrasound guidance?	
6. What are the pitfalls or artifacts using ultrasound that we should be aware of?	
7. Are there any techniques for aiding ultrasound guidance?	

## Search strategy

We broadly searched for all types of articles in the PubMed and Medline bibliographic databases from 1970 to 2020 using the following term combination in the title/abstract: “ultrasound or ultrasonography or ultrasonographically or peripheral,” “artery or arterial or vein or venous or intravenous or vascular or intravascular or vessel,” and “catheterization or cannulation or insertion or placement or access or catheter or needle or line or cannula.” A total of 23,982 articles were identified, 270 of which were found to be duplicates.

The first two authors independently screened the title/abstract of the identified articles, primarily related to vascular catheterization, with the potential to answer the seven questions. We limited the selection to 1057 articles. Thereafter, we excluded articles focusing on central venous catheter insertion, peripheral inserted central catheter insertion, midline catheter insertion, femoral arterial access, and axillary arterial access except for those referring to ultrasound approaches, techniques, pitfalls, or artifacts. A total of 384 full-text articles were assessed for eligibility, and 38 were finally included. Four additional articles were identified from the references.

## Where is the possible access site?

### Peripheral arterial catheterization

#### Arteries in the upper extremities

Peripheral arterial access sites include radial, brachial, dorsal pedis, ulnar, posterior tibial, and superficial temporal arteries [7]. The radial and ulnar arteries are the two major branches of the brachial artery and supply blood to the forearm and the hand [8]. Ultrasound-guided peripheral arterial catheterization has been studied mostly in the radial artery of the forearm because it generally has a collateral circulation with the ulnar artery [8], has less anatomic variation [8], and is associated with a low incidence of complications [7]. The most common complication of radial artery catheterization is temporary occlusion (mean incidence 19.7%). Permanent occlusion appears to be rare (0.04%) [7]. Hematoma is a common minor complication [7], and ultrasound guidance may decrease the incidence of hematoma in pediatric patients [3]. Allen’s test is commonly used to evaluate collateral blood flow in the hand before radial arterial catheterization in adults. However, in pediatric patients with congenital hand abnormalities, Allen’s test and magnetic resonance angiography of the forearm were consistent in determining patency of the palmar arch in 62% of the cases, but the sensitivity was only 28% [9].

Numerous studies have confirmed the utility of ultrasound guidance at this site in pediatric and adult patients [2, 3]. One study assessed the factors affecting catheterization success and revealed that the depth from the skin surface to the artery was strongly related to initial and overall success rates [10]; catheterization was faster and more reliable when the radial artery was 2 to 4 mm below the skin surface than < 2 mm and > 4 mm.

To our knowledge, there are no strong recommendations of ultrasound guidance for ulnar, brachial, and superficial temporal artery catheterization. However, one previous retrospective study reported that ischemic and infectious complications of the ulnar artery were as low as those of the radial artery [11]. Although further prospective studies are needed, the ulnar artery may be an alternative site when attempts at other sites prove unsuccessful.

#### Arteries in lower extremities

The posterior tibial and dorsal pedis arteries may be possible alternative access sites to the radial artery in pediatric patients without major complications [7, 12–14]. A study comparing the anatomical characteristics and utility of ultrasound-guided catheterization using the long-axis in-plane (LAX-IP) approach among the radial, posterior tibial, and dorsal pedis arteries in small children found that the posterior tibial artery is a reasonable alternative access site for ultrasound-guided radial arterial catheterization [15] because the first-attempt success rate, catheterization time, and arterial diameter of the posterior tibial artery are similar to those of the radial artery. In contrast, the dorsal pedis artery was associated with a lower first-attempt success rate, longer catheterization time, and smaller arterial diameter than the radial and posterior tibial arteries.

### Peripheral venous catheterization

#### Veins in the upper and lower extremities

The most common peripheral intravenous access site is the dorsum of the hands or feet [16]. However, veins here are sometimes invisible and impalpable in pediatric patients, and 8–50% of catheterization attempts are associated with difficult venous access [17]. Venous diameter is an important independent predictor of catheterization success [18]. Thus, the saphenous vein at the level of the medial malleolus [16] or cephalic vein at the forearm [18] has been suggested as a more preferable site for ultrasound guidance than the dorsum of the hands and feet due to its larger diameter. In adult patients, both very superficial (< 0.3 cm) and very deep (> 1.5 cm) veins are difficult to cannulate than vessels at a depth in-between [19]. However, an optimal depth for ultrasound venous catheterization in pediatric patients has not been proposed. The saphenous vein at the level of the medial malleolus or cephalic vein at the forearm is generally slightly deeper than that of the dorsum of the hands and feet [16, 18]. This may increase catheterization success with ultrasound guidance because it provides more space to adjust the needle tip to the target vessels [10, 18] and to increase image quality [16].

Veins at the cubital fossa are impalpable in most cases [16] and are generally considered to have sufficient venous diameter and depth because they are located upstream of the cephalic vein at the forearm. Thus, they could be alternative access sites in cases of prior unsuccessful catheterization in other areas. However, there appear to be no reports of ultrasound-guided catheterization in these cases, and there is also a risk of inadvertent puncture of the brachial artery [16]. Furthermore, the catheter may not work well when the elbow is bent.

## How do we check the vascular condition?

One of the advantages of ultrasound-guided vascular catheterization is that the distinction between arteries and veins and the patency of the vessels can be confirmed by Doppler mode imaging achieved using the following techniques: (1) lightly squeezing the blood vessels with a probe under the color Doppler mode, (2) viewing the direction of blood flow under the color Doppler mode, and (3) distinguishing between arteries and veins using the wave pattern of the pulse wave Doppler mode [20, 21]. Furthermore, combining these techniques may increase accuracy. Peripheral veins have a lower blood flow velocity than arteries, and spontaneous signals may not be generally detected in the color Doppler mode. In these cases, distal compression allows the squeezing of the blood from the vein, elevating blood flow velocity, and consequently aiding in the demonstration of flow in patent veins [21].

## How do we obtain the optimal position for the procedure?

The position has been shown to alter arterial anatomical characteristics. In adult patients, the location and patency of the radial artery differed according to wrist position [22, 23], and attempts at a 45° wrist angle might be advantageous in an ultrasound-guided LAX-IP approach to radial artery cannulation in relation to cannulation time and first-attempt success rate [22]. In pediatric patients, differences in anatomical characteristics among the radial, posterior tibial, and dorsal pedis arteries under the influence of modified positions (such as wrist dorsiflexion up to 45° in the radial artery, ankle dorsiflexion and eversion in the posterior tibial artery, and ankle plantar flexion in the dorsal pedis artery) have been investigated [15]. The depth from the skin surface was found to be shallower, and the arterial diameter did not significantly alter after changing to the modified positions in the three arteries. Interestingly, the cross-sectional area was slightly lower in the posterior tibial and dorsal pedis artery under the influence of modified positions but did not significantly change in the radial artery. Further research is needed to determine the effect of position change on catheterization success in these arteries.

## How do we determine the optimal catheter size?

The determination of catheter size should be based on (1) the relationship between vessel and catheter diameter and (2) the catheter travel distance from skin to vessel. Larger arterial catheters may provide better tracings, easier blood sampling, and longer patency than smaller arterial catheters. However, comparing the use of 18G and 20G catheters in adult patients, the incidence of arterial occlusion after 24 h of cannulation increased linearly, as a larger vessel lumen area was occupied by the catheter [24]. Furthermore, if the catheter occupied up to 20% of the arterial lumen, the occurrence of occlusion was rare.

In pediatric settings, a 24G or 22G catheter, with a diameter of approximately 0.7 or 0.9 mm, respectively, is commonly used. Assuming that the artery is round, a minimum arterial diameter of 1.57 mm is required to not exceed 20% of the arterial lumen when using a 0.7-mm catheter. However, arterial diameter is often below the required diameter, especially in infants and small children, ranging approximately from 0.7 to 1.7 mm [10, 15]. Thus, measuring the internal diameter of the radial artery in young children prior to ultrasound-guided cannulation will ensure a more appropriate selection of catheter size [25].

In clinical settings that may require large amounts of transfusion or blood transfusion, larger venous catheters are preferably used. However, a relatively larger catheter compared with venous diameter is reported as a risk factor of infiltration, a major complication of venous catheterization [26]. Regarding the prevention of infiltration, the catheter diameter should be less than 30% of the venous lumen [27].

A longer catheter travel distance from skin to vessel is associated with catheter failure, especially dislodgement. A study investigating catheter survival in ultrasound-guided peripheral venous catheterization in adults reported that 100% of intravenous catheters failed when < 30% of the entire length of the catheter was in the vein, 32.4% of intravenous catheters failed when 30–64% of the catheter was in the vein, and no intravenous catheters failed when ≥ 65% of the catheter was in the vein [28]. Furthermore, the risk of catheter failure decreased by 29% for every 5% increase of catheter length located in vein. This evidence is supported by another study investigating the survival of an ultralong peripheral catheter in adults than a standard-length catheter in ultrasound guidance; it reported a markedly higher catheter survival rate when the catheter length in the vein exceeded 2.75 cm [29].

Given that the insertion angle is actually between approximately 30° and 45° when performing catheterization in pediatric settings [10, 15, 30, 31], the minimum required catheter length is presumed to be the catheter travel distance from skin to vessel, which is the perpendicular distance corrected by the actual insertion angle (Additional file 1A). An insertion angle of 45° is the main and most convenient way to presume approximate catheter travel distance because it is calculated as the perpendicular depth × 1.4 using the Pythagorean theorem (Additional file 1B) Therefore, the catheter travel distance is 0.7 cm when approaching a vessel of 0.5 cm perpendicular depth at an insertion angle of 45°, which requires a catheter length of 2.0 cm under the presumption that 65% of the catheter length is inside the vessel. If the insertion angle is set at less than 45°, the minimum required catheter length will be longer. A standard-length 24G or 22G catheter is approximately 19 mm or 25 mm, respectively. Thus, when approaching vessels that are deeper than 0.5 cm below the skin surface, longer catheters should be considered.

## What are the major approaches available for ultrasound guidance?

LAX and short-axis (SAX) views are the two major planes of target vessel visualization in ultrasound guidance. In the LAX and SAX views, the ultrasound intersects the target vessel longitudinally and vertically, respectively. Regarding the catheter, there are also two major ultrasound planes used for visualization. With the IP approach, the catheter moves on the ultrasound plane and the whole catheter shaft is visualized as it advances toward the vessel. With the out-of-plane (OOP) approach, the catheter and ultrasound plane are at right angles to each other and only the needle tip is visualized on the screen.

**Fig. 1:**
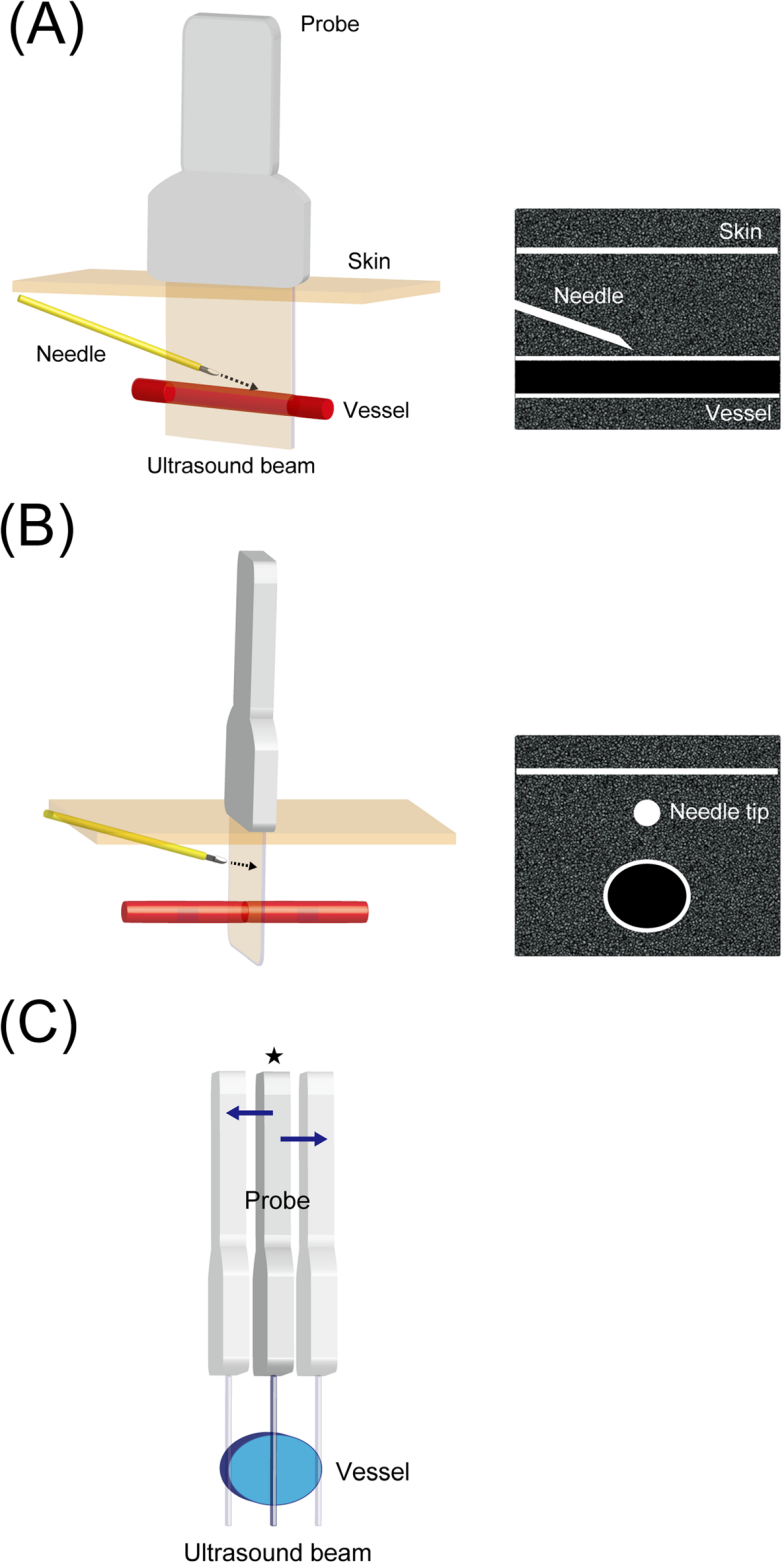
Schematics and ultrasound views of the long-axis in-plane (LAX-IP) and short-axis out-of-plane (SAX-OOP) approaches are shown in **a** and **b**, respectively. In the LAX-IP approach, the whole shaft of the needle and the entire course of the target vessel are constantly on the screen during the procedure. In the SAX-OOP approach, the target vessel is visualized in transverse orientation and only the needle tip is traced as a high-echoic point with acoustic shadow on the ultrasound image. The main potential disadvantage of the long-axis in-plane (LAX-IP) approach in pediatric settings (**c**). In cases approaching small vessels, the probe is easily moved away from the plane at which the ultrasound beam intersects the target vessels at the largest diameter (★)

Of these plane combinations, LAX-IP and SAX out-of-plane (SAX-OOP) approaches are used for ultrasound guidance (Fig. 1a, b) [32]. In a recent systematic review and meta-analysis of live adult patients and phantoms, the SAX-OOP approach had a higher success rate than the LAX-IP approach [33]. In pediatric patients, controversy remains regarding whether the LAX-IP or SAX-OOP approach is superior in PVC [34, 35]. Thus, operators should be familiar with the advantages and disadvantages of each approach.

### Long-axis in-plane approach

In the LAX-IP approach, the whole shaft of the needle and the entire course of the target vessel remain on the screen during the procedure for ultrasound guidance in “real time,” which is advantageous in terms of avoiding complications [36]. However, when approaching small vessels, it is difficult to maintain the probe along the best plane in which the largest diameter is visualized along its course. Attempts other than at maximum diameter may easily result in failure when the catheter and vessel diameter are similar. Thus, this may be the main disadvantage of the LAX-IP approach in pediatric settings. (Fig. 1c).

### Short-axis out-of-plane approach (static approach)

In the SAX-OOP approach, only the needle tip is visualized as a high-echoic point with acoustic shadow on ultrasound images, and the remaining shaft is off screen. Briefly, after puncturing the anterior wall at the maximum diameter and confirming the needle tip in the vessel and back flow in the catheter hub, the operator advances the needle slightly with a reduction in insertion angle and places the catheter without ultrasound guidance. The major advantage of the SAX-OOP approach, particularly in pediatric patients, is that the maximum vessel diameter can be constantly visualized until puncturing the anterior wall. However, due to the process being performed without ultrasound guidance, the SAX-OOP approach is considered inferior to the LAX-IP approach regarding “real time.” Additionally, accidental posterior-wall penetration during the procedure off screen is considered the main disadvantage of the SAX-OOP approach. For these reasons, the SAX-OOP approach is referred to as the “static approach” in contrast to “dynamic needle tip positioning” (DNTP), described below.

### Dynamic needle tip positioning

DNTP was first described in 2012 and is an alternative derived from the “static” SAX-OOP approach [37]. In DNTP, the operator traces the needle tip by sweeping the probe akin to making a flip cartoon of the catheterization in the SAX-OOP approach.

**Fig. 2:**
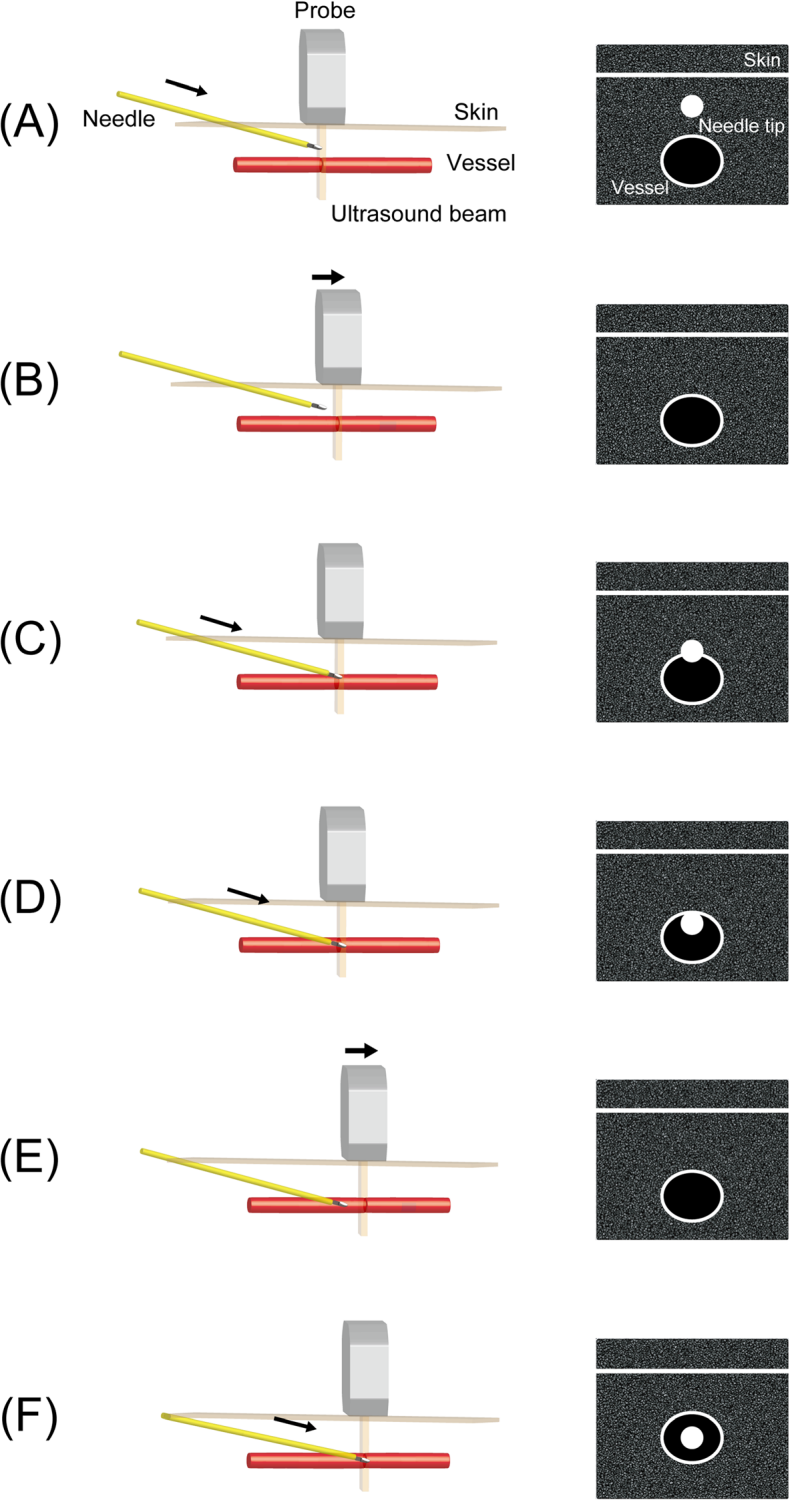
Dynamic needle tip positioning schematic. **a** The needle tip is visualized as a high-echoic point between the skin surface and the anterior wall of the target vessel on the ultrasound image. **b** The probe is advanced toward slightly until the high-echoic point disappears from the image. **c** The needle tip is advanced further toward the anterior wall, and the high-echoic point is visualized just over the target vessel. **d** The needle tip penetrates the anterior wall under ultrasound guidance, and the high-echoic point is visualized in the target vessel. **e** The probe is further advanced until the high-echoic point disappears from the image. **f** The needle tip is advanced further slightly with a reduced puncture angle until the high-echoic point appears on the image


The approach is briefly described as follows. (1) The needle tip is visualized as a high-echoic point between the skin and the anterior vessel wall at the maximum diameter in the plane. (2) While holding the needle in position, the probe is advanced slightly forward along the travel course of the catheter until the high-echoic point disappears from the plane. (3) While holding the probe in position, the needle is advanced further until the high-echoic point reappears in the plane. Steps 1, 2, and 3 are repeated alternately until it is safe to thread off the catheter (at least until the whole needle tip is inserted into the vessel) (Fig. 2a–f and Additional file 2). Thus, due to DNTP providing a much better tracing of the needle tip, it is considered to complement the SAX-OOP approach in terms of “real time.” To support this, DNTP has been shown to have a greater first-attempt (peripheral venous catheterization, 86.7 vs. 60%; arterial catheterization, 85 vs. 50%) and overall success rate (peripheral venous catheterization, 90 vs. 63.3%; arterial catheterization, 95 vs. 60%) in ultrasound-guided vascular catheterization in pediatric patients than the “static” SAX-OOP approach [31, 38]. However, as described above, DNTP consists of a series of precise techniques, and sufficient experience in ultrasound guidance is required to adequately perform this procedure in smaller children [39].

**Additional file 2** Dynamic Needle Tip Positioning.

## What are the pitfalls or artifacts using ultrasound that we should be aware of?

**Fig. 3:**
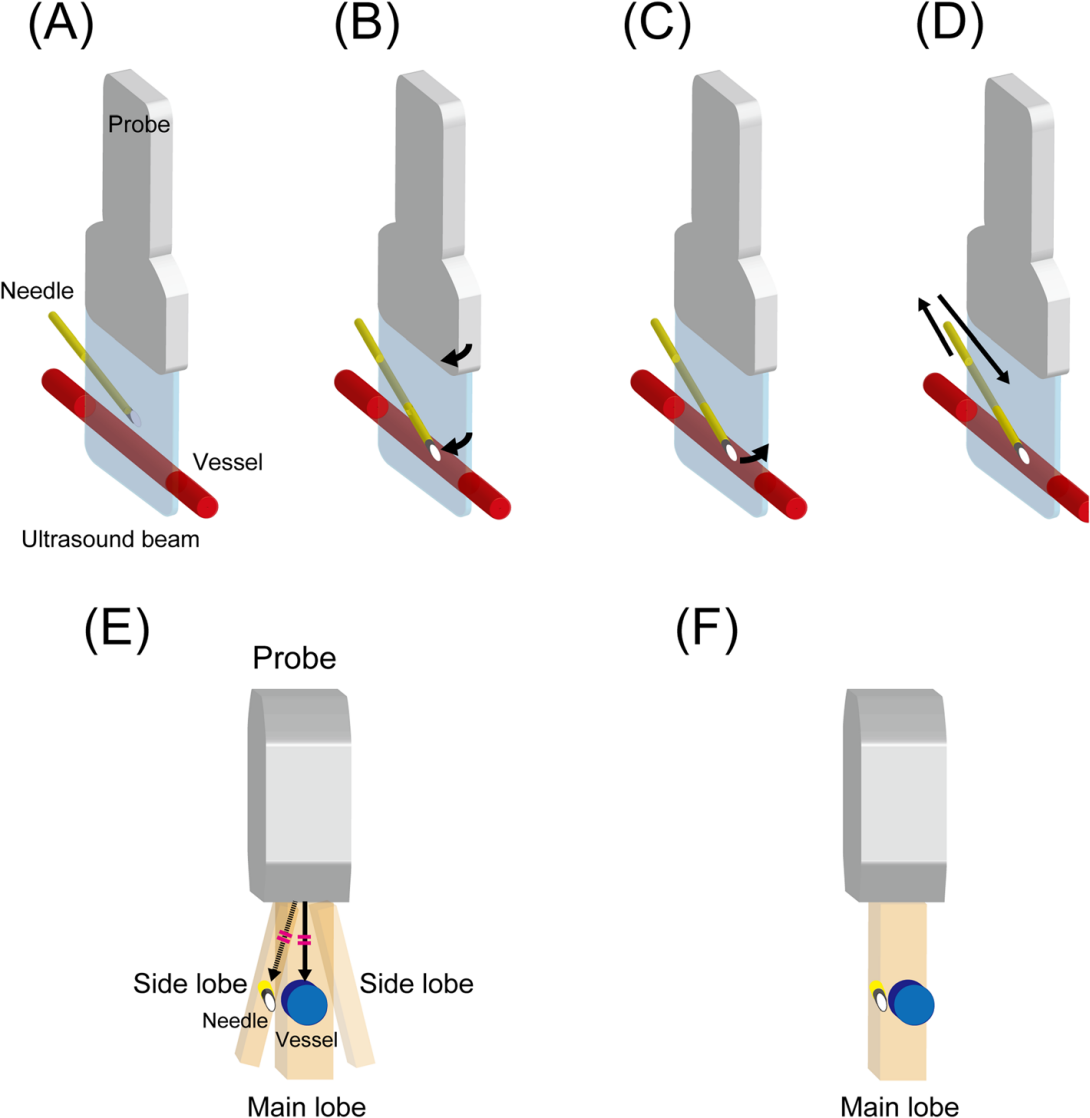
Schematic shows the possible technique options for returning the needle into the ultrasound plane in the LAX-IP approach when the needle leaves the plane as it advances. **c** and **d** may be more suitable for pediatric settings because the ultrasound plane does not move away from the “best plane.” **a** The needle is on the ultrasound plane. **b** The probe is slightly rotated toward the catheter. **c** The catheter is slightly moved toward the center line of the probe. **d** The catheter is withdrawn to the near skin, and the procedure is restarted. Side-lobe and slice-thickness are possible explanations for cases when the catheter appears to be placed successfully into the vessel in the LAX-IP approach, but no back flow is observed in the catheter hub. The two artifacts are very similar [40]. **e** When a strong reflector occurs in the side-lobe beam, the ultrasound machine mixes the reflected signals of the main and side-lobe beam, and structures these signals into the same ultrasound image (side-lobe artifact). **f** The ultrasound machine assumes that the emitted beam is extremely thin. However, the beam actually has a measurable thickness that varies with depth. Thus, when the needle and the vessel are in the same beam width, even if the needle is not inserted into the vessel, they are structured into the same ultrasound image (slice-thickness artifact)

In the LAX-IP approach, the needle, especially its tip, sometimes leaves the ultrasound plane as it advances. To return the needle into the plane, the operator can perform one of the following: (1) slightly rotate the probe toward the catheter, (2) move the catheter toward the center line of the probe, or (3) withdraw the catheter to near the skin and restart the procedure (Fig. 3a–d). The latter two may be more suitable for pediatric settings because the ultrasound plane does not move away from the “best plane.”

Given that the LAX-IP approach is prone to side-lobe or slice-thickness artifacts [10, 40–42], the catheter can appear to be in the same plane as the vessel even when the catheter has not successfully cannulated the vessel (Fig. 3e, f). The operators should consider the possibility of this phenomenon when the entire catheter looks to be placed successfully into the vessel in the LAX-IP approach, but no back flow is observed in the catheter hub.

**Fig. 4:**
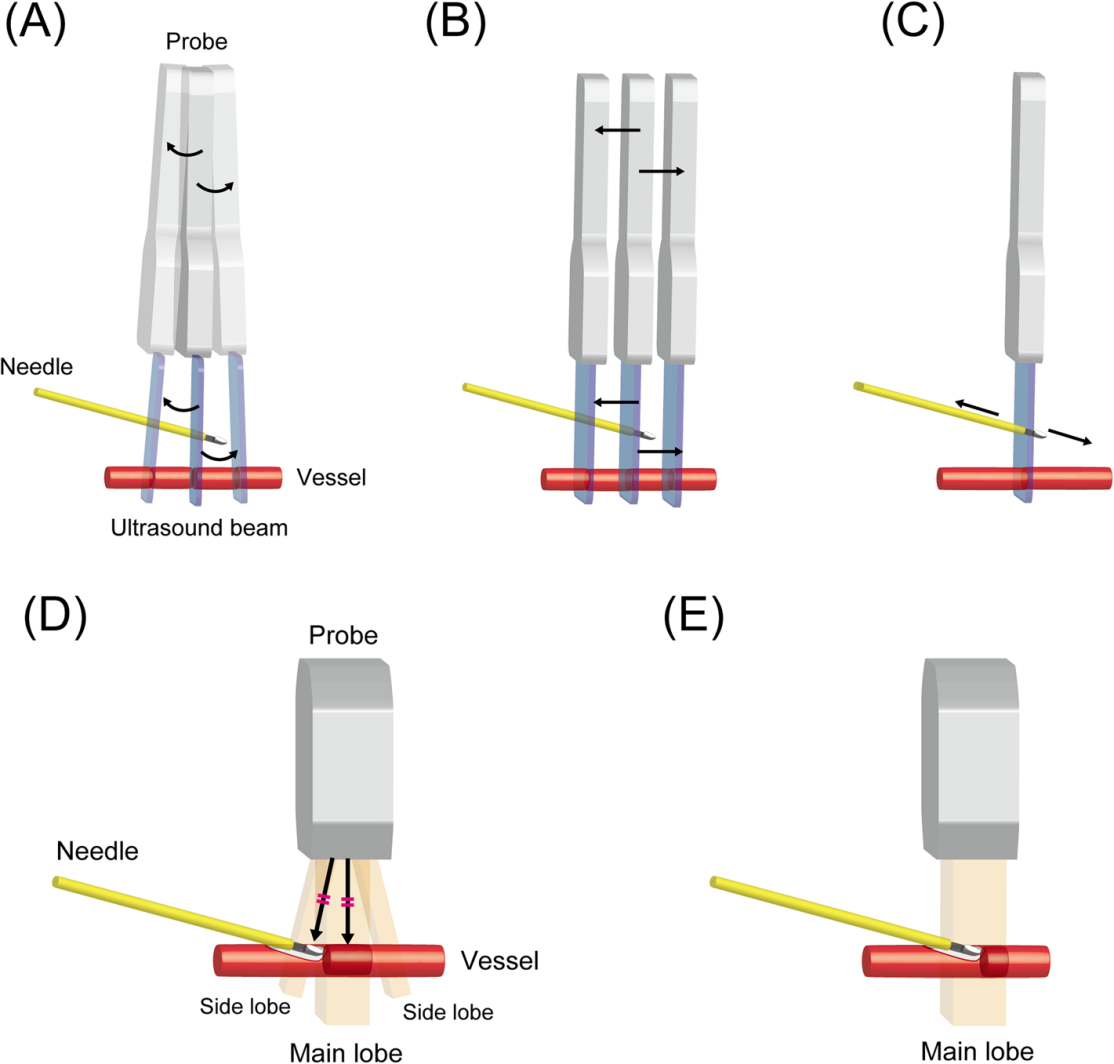
Several technique options for confirming the needle tip in the short-axis out-of-plane (SAX-OOP) approach. **a** Tilting the probe back and forth along the travel direction of the catheter. **b** Sweeping the probe back and forth along the travel direction of the catheter. **c** The catheter can be moved back and forth while straddling the ultrasound plane when the operator does not want to move away from the best plane. The following are possible explanations of when the needle tip looks to be placed successfully into the vessel in the SAX-OOP approach, but no back flow is observed in the catheter hub. **d** When a strong reflector is in the side-lobe beam, the ultrasound machine mixes the reflected signals of the main and side-lobe beam, and structures these signals into the same ultrasound image (side-lobe artifact). **e** The ultrasound machine assumes that the emitted beam is extremely thin. However, the beam actually has a measurable thickness that varies with depth. Thus, when the needle tip and the vessel are in the same beam width, even if the needle tip is in the vessel, they are structured into the same ultrasound image (slice-thickness artifact)

Compared with the LAX-IP approach, it is more difficult for the operator to recognize that the needle tip leaves the plane in the SAX-OOP approach because the shaft is also visualized as a high-echoic point on the ultrasound image. When the needle tip exceeds the ultrasonic plane, the high-echoic point caused by the shaft mimics that by the needle tip. There are several ways to confirm the needle tip in the SAX-OOP approach. Techniques that can adjust the probe include tilting or sweeping back and forth along the travel direction of the catheter (Fig. 4a, b). When the operator wants to avoid moving the probe when maintaining the best plane, the catheter can be confirmed with the appearance and disappearance of the high-echoic point on the screen alongside slightly moving it back and forth (Fig. 4c).

In the SAX-OOP approach, the back flow may not be confirmed in the catheter hub when the needle tip appears to be successfully placed in the vessel. The potential eventuality is that the needle does not puncture but instead presses the anterior wall of vessel due to elasticity (Additional file 3), and side-lobe or slice-thickness artifacts can occur (Fig. 4d, e) [10, 40–42]. In these cases, the operator has a chance to insert the needle tip into the true vessel lumen by further advancing the needle. However, the operator should consider the possibility of posterior-wall penetration when no back flow is observed with further advancement of the needle.

Posterior-wall penetration due to puncture pressure from the needle can still occur in DNTP. In most such cases, the kissing of the anterior and posterior walls under pressure can be visualized before puncturing the vessel (Additional file 4). When posterior-wall penetration is suspected, the operator can trace the needle tip, with further slight advancing or tilting of the probe, to determine whether it is visualized below the posterior wall. If it penetrates the posterior wall, the operator can restore the position of the probe and withdraw the needle tip until it reappears in the center of the vessel and a continuous back flow is observed (“reverse” DNTP). Then, the narrowed vessel lumen is generally widened again by the force of withdrawing the needle and the operator can restart “forward” DNTP.

## Are there any techniques aiding ultrasound guidance?

For pediatric patients in whom the radial artery was located at a depth of < 2 mm, increasing the depth to 2–4 mm via a subcutaneous saline injection improved the catheterization time and success rate in the SAX-OOP approach [10]. Saline injection also provided an anechoic area on the anterior arterial wall, which enhanced the ultrasound signals and improved the visibility of the anterior arterial wall and needle tip (Additional file 5).

The technique using the correspondence of the middle mark on the probe and the ultrasound image may facilitate needle placement in DNTP and has been termed “modified DNTP” [5]. The utility of this modified approach in peripheral radial arterial catheterization was investigated in neonates by experienced operators and was shown to improve the first-attempt and total success rates and decrease total procedural time and incidence of cannulation-related complications [5]. However, in this challenging patient population, difficulty remains in recognizing the accurate location of the needle tip because the artery is very superficial, generally at a depth of < 2 mm, and the ultrasound provides a stronger echo on the screen. To resolve this, modified DNTP combined with saline injection was proposed and investigated in neonates by trainees [39]. Therefore, modified DNTP combined with saline injection had a higher success rate within 10 min (72.9 vs. 47.9%), shorter median catheterization time (203 vs. 600 s), and lower incidence of hematoma postoperatively (18.3 vs. 22.9%) than that without saline injection.

## Limitations

The sample size of some of the studies was relatively small. We included observational studies and case reports, especially for answering the question regarding access sites. Thus, the quality of some of the evidence is variable, and the answers may differ when higher quality data become available. Catheter size determination is mainly based on the findings in adults. However, preventing infiltration and catheter failure through proper catheter selection is an important clinical issue, and these considerations may provide some guidance even in pediatric settings.

## Conclusion

There is accumulating evidence supporting the utility of ultrasound guidance, and its application for peripheral vascular access in pediatric patients is becoming increasingly widespread. Operator experience and use of appropriate techniques based on a systematic understanding of the basics are necessary to ensure successful ultrasound-guided peripheral vascular catheterization and the prevention of complications.

## Supplementary information

**Additional file 1.** Required catheter length presumption by the insertion angle. (A) Minimum catheter travel distance from skin to vessel should be calculated from the perpendicular distance corrected by the actual insertion angle. (B) Presumption of an insertion angle of 45° is the most common and convenient way to determine approximate catheter travel distance (Pythagorean theorem).

**Additional file 3.** Representative ultrasound image of the vessel wall tenting. An example of when the needle does not puncture but presses the anterior wall of the vessel due to elasticity. Ultrasound-guided internal jugular venous catheterization of the long-axis in-plane approach in a pediatric patient is provided for improved visualization.

**Additional file 4.** Representative ultrasound image of the vessel walls kissing by puncture pressure. A narrower vessel lumen (the kissing of the anterior and posterior walls, white arrow) under puncture pressure before the puncturing of the anterior wall.

**Additional file 5.** Representative ultrasound images of saline injection method. (A) Radial artery located at a depth of 1.3 mm from the skin surface (white arrow). (B) Saline injection increases the depth from 1.3 mm to 2.6 mm. Furthermore, it provides an anechoic area on the anterior arterial wall, which enhances the ultrasound signals and improves the visibility of the anterior arterial wall and needle tip. (★).

## Data Availability

Not applicable

## Abbreviations

LAX: Long-axis
SAX: Short-axis
IP: In-plane
OOP: Out-of-plane
LAX-IP: Long-axis in-plane
SAX-OOP: Short-axis out-of-plane
DNTP: Dynamic needle tip positioning
