# Supplementary figures and images for: Ultrasound-guided peripheral vascular catheterization in pediatric patients: a narrative review
Source: Crit Care. 2020 Sep 30;24:592. doi: 10.1186/s13054-020-03305-7 (PMC7526377; doi:10.1186/s13054-020-03305-7)

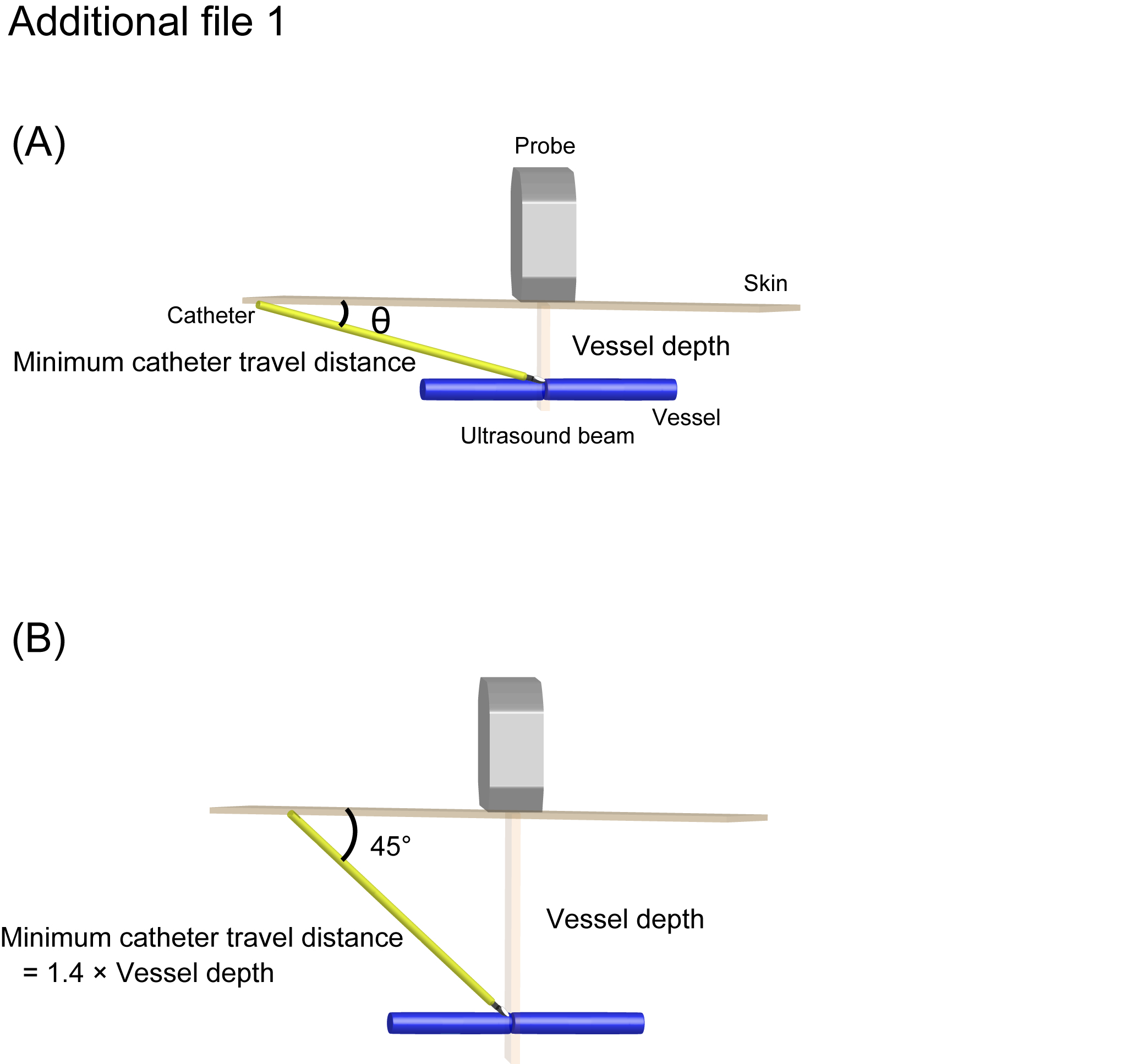

Supplement: Supplementary file 2 — Additional file 1. Required catheter length presumption by the insertion angle. (A) Minimum catheter travel distance from skin to vessel should be calculated from the perpendicular distance corrected by the actual insertion angle. (B) Presumption of an insertion angle of 45° is the most common and convenient way to determine approximate catheter travel distance (Pythagorean theorem). [file 13054_2020_3305_MOESM1_ESM.jpg]

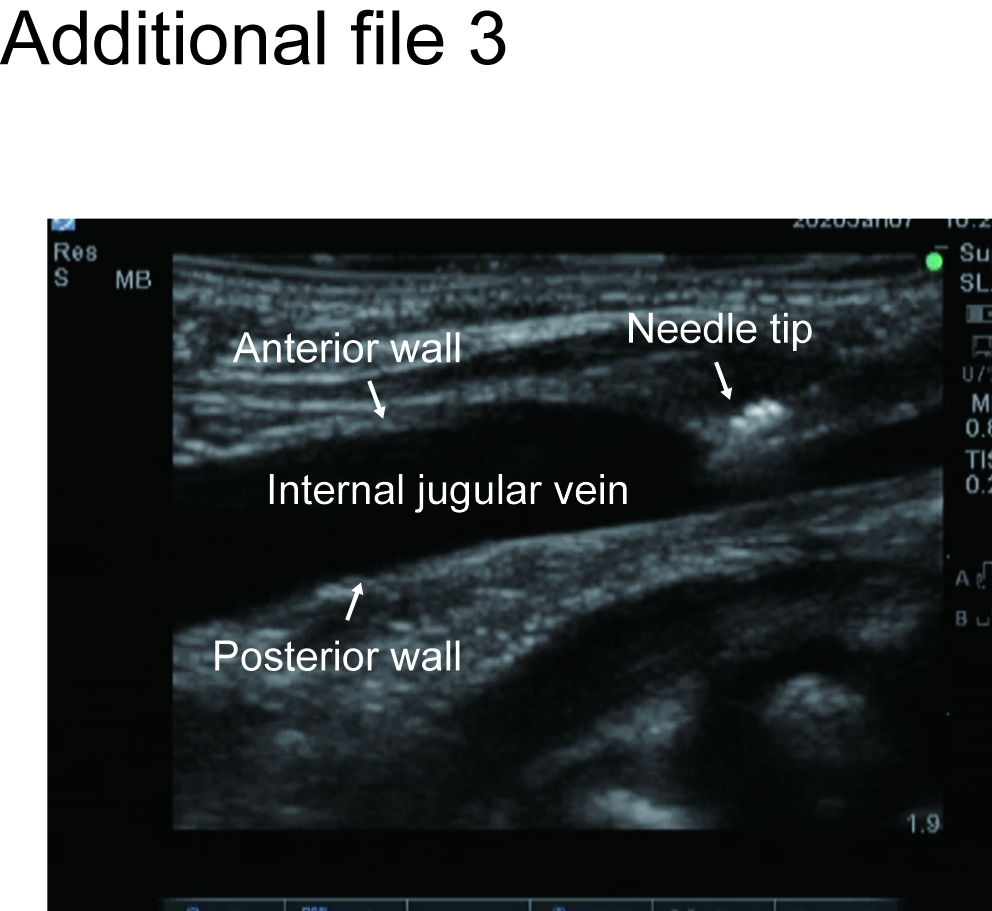

Supplement: Supplementary file 3 — Additional file 3. Representative ultrasound image of the vessel wall tenting. An example of when the needle does not puncture but presses the anterior wall of the vessel due to elasticity. Ultrasound-guided internal jugular venous catheterization of the long-axis in-plane approach in a pediatric patient is provided for improved visualization. [file 13054_2020_3305_MOESM3_ESM.jpg]

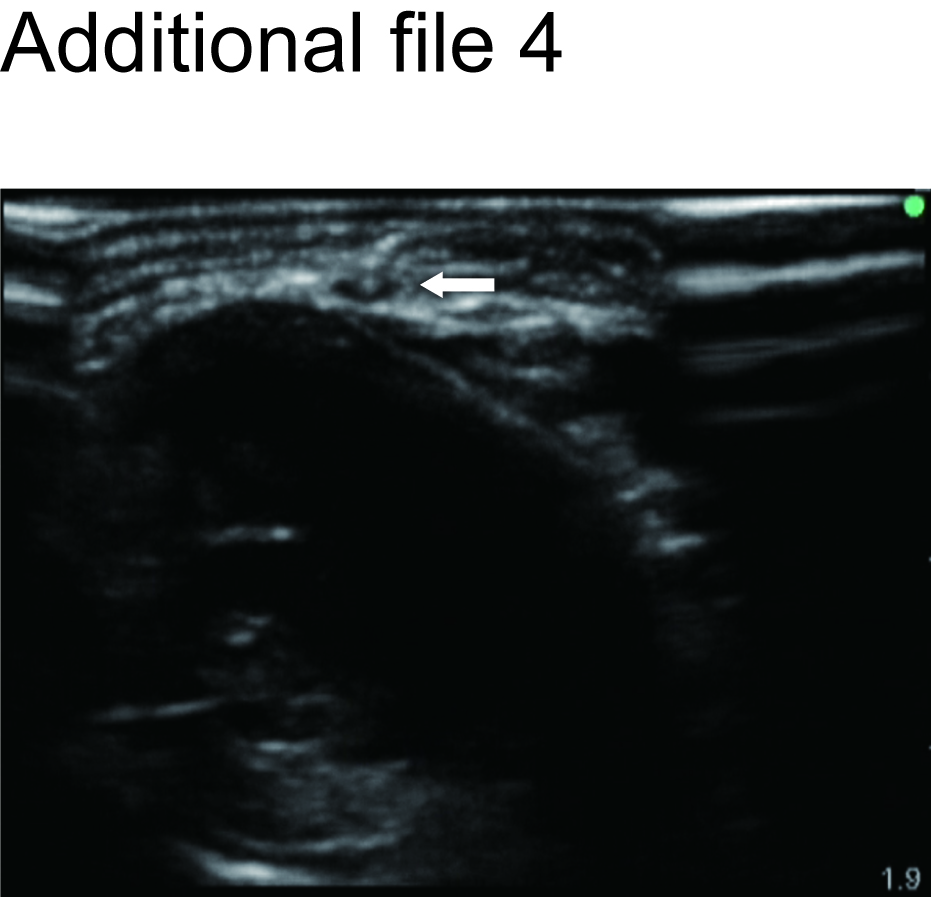

Supplement: Supplementary file 4 — Additional file 4. Representative ultrasound image of the vessel walls kissing by puncture pressure. A narrower vessel lumen (the kissing of the anterior and posterior walls, white arrow) under puncture pressure before the puncturing of the anterior wall. [file 13054_2020_3305_MOESM4_ESM.jpg]

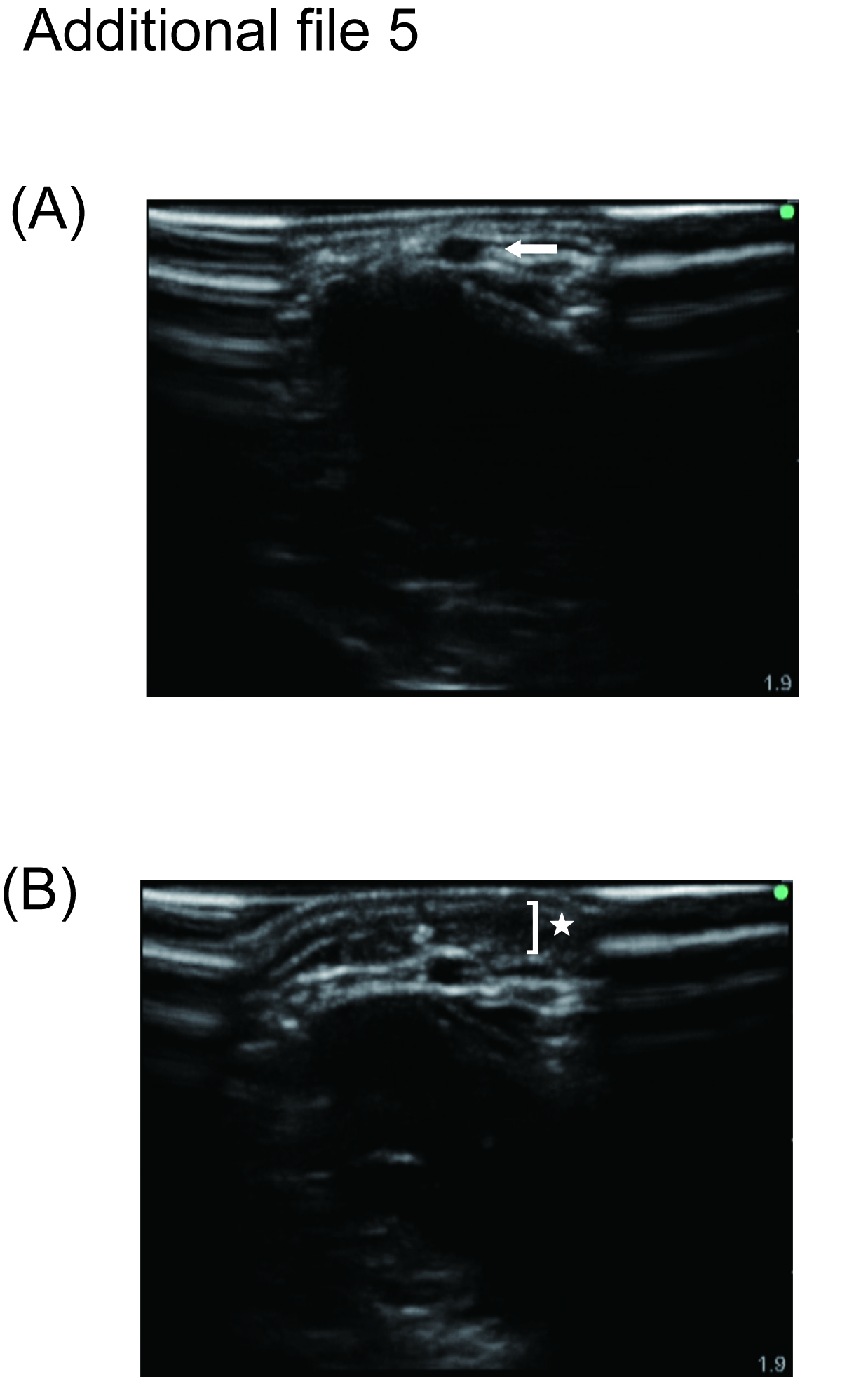

Supplement: Supplementary file 5 — Additional file 5. Representative ultrasound images of saline injection method. (A) Radial artery located at a depth of 1.3 mm from the skin surface (white arrow). (B) Saline injection increases the depth from 1.3 mm to 2.6 mm. Furthermore, it provides an anechoic area on the anterior arterial wall, which enhances the ultrasound signals and improves the visibility of the anterior arterial wall and needle tip. (★). [file 13054_2020_3305_MOESM5_ESM.jpg]
